# Supplementary material for: Polarons Explain Luminescence Behavior of Colloidal Quantum Dots at Low Temperature
Source: Sci Rep. 2018 May 30;8:8385. doi: 10.1038/s41598-018-26678-w (PMC5976793; doi:10.1038/s41598-018-26678-w)
Supplement: Supplementary file 1 — Supplementary Information [file 41598_2018_26678_MOESM1_ESM.pdf]

Supplementary Information

Polarons Explain Luminescence Behavior of Colloidal Quantum Dots at Low Temperature

Meenakshi Khosla, Sravya Rao, and Shilpi Gupta

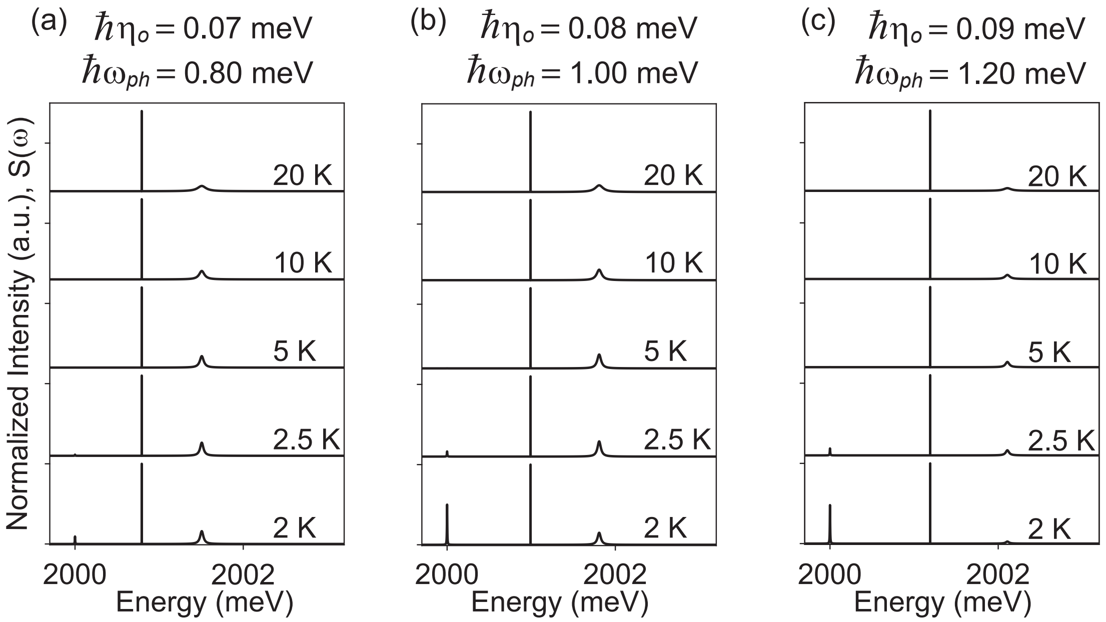

Figure S 1. Spectrum for different values of ω_{ph} and η_0. (a)$\hbar\eta_0 = 0.07$ meV and $\hbar\omega_{ph} = 0.80$ meV. (b)$\hbar\eta_0 = 0.08$ meV and $\hbar\omega_{ph} = 1.00$ meV. (c)$\hbar\eta_0 = 0.09$ meV and $\hbar\omega_{ph} = 1.20$ meV. Spectral diffusion is not included.

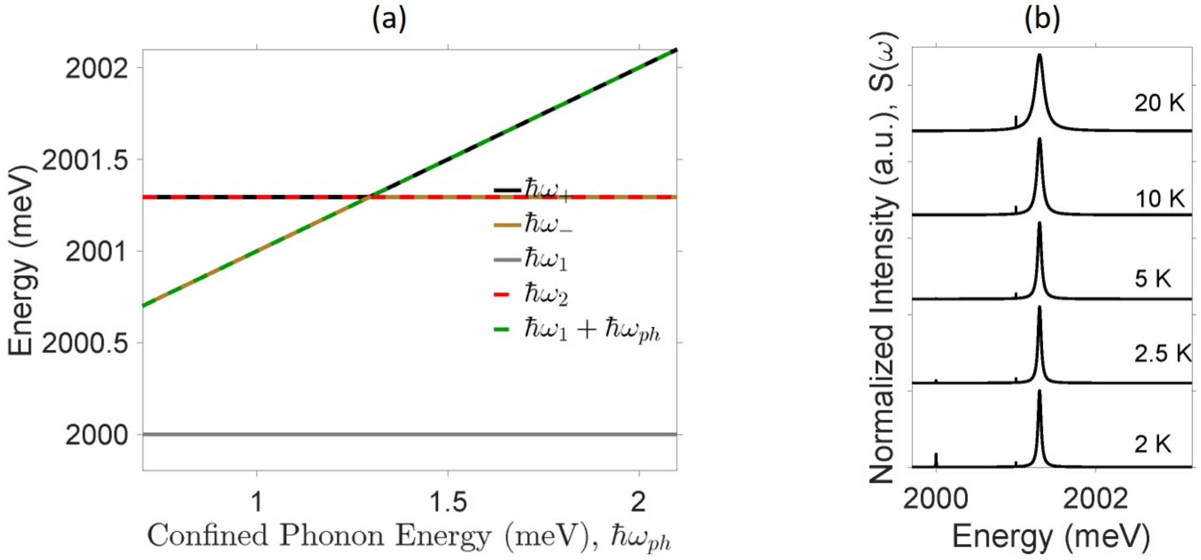

Figure S 2. Spectrum when strong coupling condition is not satisfied ($\hbar\eta_0 = 0.001meV$). (a) Energy levels not showing anti-crossing behavior (b) Resulting spectrum does not show experimentally observed spectral behavior. Spectral diffusion is not included.

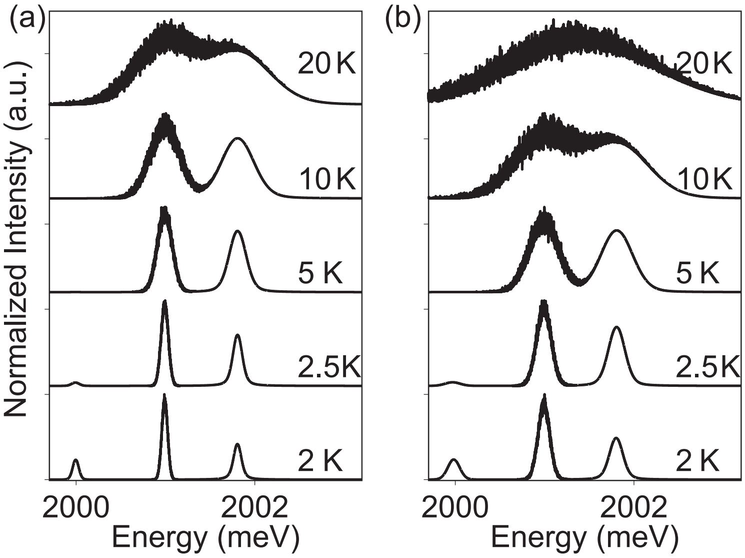

Figure S 3. Spectrum including spectral diffusion as a Gaussian distribution with standard deviation varying linearly with temperature T. Standard deviation (a) $4T$ ns^{-1}. (b) $8T$ ns^{-1}.

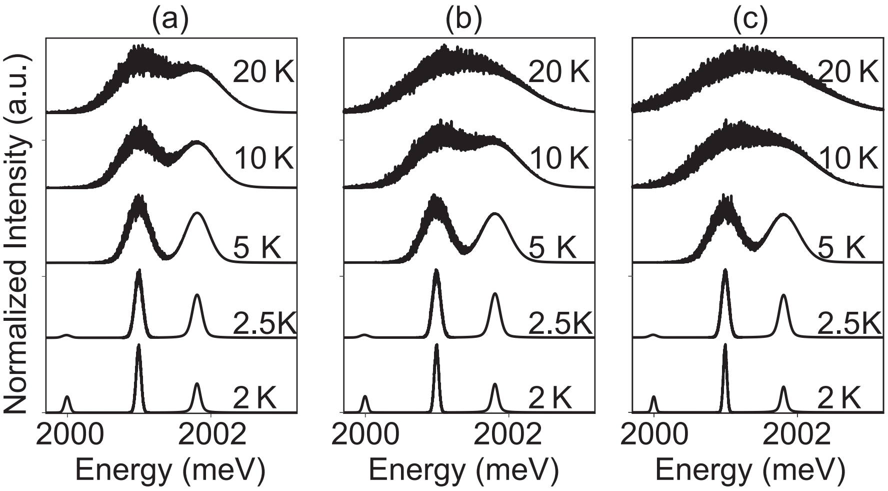

Figure S 4. Spectrum including spectral diffusion as a Gaussian distribution with standard deviation varying with temperature T as Boltzmann Distribution. Standard deviation (a) $100e^{-5/T}$ ns^{-1}. (b) $150e^{-6/T}$ ns^{-1}. (c) $200e^{-7/T}$ ns^{-1}.

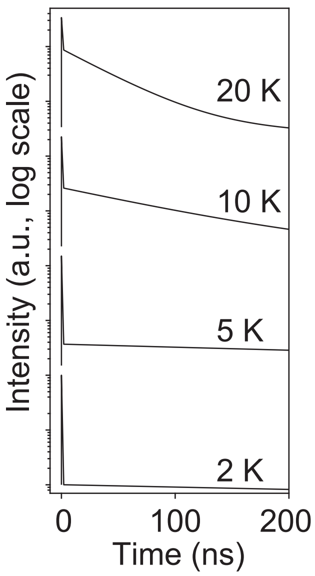

Figure S 5. Decay curves for a different set of initial conditions. $\rho_{++}(0) = 0.5, \rho_{--}(0) = 0.5, \rho_{11}(0) = 0$.

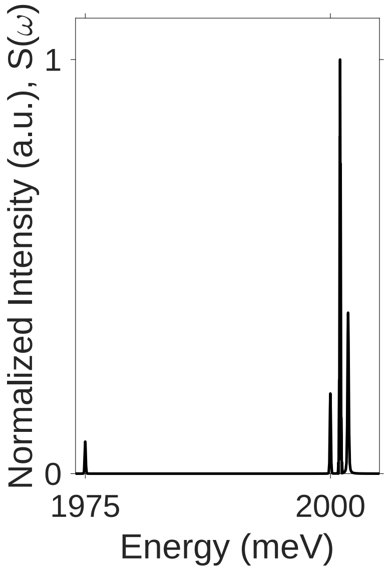

Figure S 6. Spectrum including LO phonon assisted decay of the pure dark state $|1>$. The scheme is similar to discussed in Wijnen et al, Phys. Rev. B, **78**, 235318 (2008). We set the rate of LO-phonon assisted decay of the pure dark state to be $0.4\Gamma_1$ and the rate of photon emission through this path to be Γ_-.
